# Tooth Graft: An Umbrella Overview

**DOI:** 10.1055/s-0043-1764420

**Published:** 2023-04-14

**Authors:** Sara Hashemi, Shivasadat Tabatabaei, Amirhossein Fathi, Seyedeh Mohadeseh Asadinejad, Ramin Atash

**Affiliations:** 1 Dental Students Research Committee, School of Dentistry, Isfahan University of Medical Sciences, Isfahan, Iran; 2 School of Public Health, Boston University, Boston, MA, USA; 3 Dental Prosthodontics Department, Dental Materials Research Center, School of Dentistry, Isfahan University of Medical Sciences, Isfahan, Iran; 4 Periodontology Department, School of Dentistry, Isfahan University of Medical Sciences, Isfahan, Iran; 5 Department of Prosthodontics, School of Dentistry, Faculty of Medicine, Université Libre de Bruxelles, Brussels, Belgium

**Keywords:** tooth graft, bone regeneration, bone substitutes, alveolar ridge augmentation

## Abstract

This umbrella review aims to evaluate systematic/meta-analysis studies containing clinical evidence on tooth grafts as bone substitutes in the oral and maxillofacial regions. Using language restrictions and Preferred Reporting Items for Systematic Reviews and Meta-Analyses (PRISMA) guidelines, an electronic database search of PubMed, MEDLINE, Embase, Cochrane library, and Google Scholar was conducted, featuring published studies up until August 2022. All systematic/meta-analysis review articles relating to tooth graft materials were matched against the inclusion criteria. Two qualified researchers independently assessed the studies' inclusion or exclusion criteria and risk of bias, and a third investigator assisted in resolving ambiguities. A total of 81 systematic/meta-analysis studies, comprising 21 animal-controlled trials, 23 randomized controlled human trials, 23 prospective studies, and 14 retrospective studies, were selected for this study. A small risk of bias was observed in systematic studies/meta-analyses. In addition, the clinical evidence from the analysis of these studies revealed a low incidence of side effects. According to the current review, two systematic reviews indicated that autogenous bone grafting of prepared teeth might be as effective as other bone grafting materials. Four studies also mentioned autologous grafts as potential alternatives to autologous grafts, autogenous demineralized dentin (ADDM), engineered grafts, root blocks, and dental matrix. On the other hand, three systematic studies stated that more long-term research is needed to confirm their findings. Finally, given the importance of standardization and homogeneity of studies for clinical cases, it is advised to be used cautiously due to the risks of transplant rejection.

## Introduction

Due to the contractile activity of myofibroblasts, the alveolar bone tends to decrease in volume following a tooth extraction, especially on the vestibular side. Therefore, preserving and regenerating alveolar bone has historically been a major challenge in implant dentistry. 1 Dental implants must be firmly stabilized with sufficient bone. Consequently, some patients would be ineligible for implant treatment without horizontal or vertical bone augmentation. 2 Vertical bone augmentation in the mandible is more challenging and less predictable than horizontal augmentation, 2 but maxilla sinus management can provide predictable conditions and satisfactory results for vertical augmentation 3 .

In the past, bone grafting materials were used to restore the degenerated bone to regenerate alveolar bone due to periodontal diseases, jaw, and facial surgical defects. Increasing advances in dental implantology have led to the development of numerous techniques and procedures, such as bone expansion and sinus floor elevation. Currently, the gold standard for reconstructing hard tissue is an autogenous bone graft. 4 Nevertheless, donor site issues, virus transmission, resorption, limited access, and the creation of surgical sites can be cited as complications associated with this technique. Due to these factors, numerous studies on bone replacement materials, such as mineralized and freeze-dried bone allografts and synthetic alloplastic grafts, have been conducted. 5 6

Using teeth as a graft material has been investigated through case reports and retrospective, prospective studies, randomized controlled trials (RCTs), and controlled clinical trials (CCTs). 7 8 9 10 11 12 The general instructions for preparing transplant materials include extraction of autogenous, allogeneic, or xenogeneic teeth, preparation of soft-tissue and tooth fragmentation, demineralization/remineralization, and sterilization. In addition, several animal studies have reported osteoconductive and osteoinductive effects using dentin as an autograft. 7 13 Some RCTs comparing tooth bone grafts to other bone substitutes found comparable outcomes, 9 10 while other studies found no difference. 14 In addition, some articles have reported negative side effects, including implant loosening, bone graft failure, and infection. 11 12

This umbrella review analyzed nine systematic reviews/meta-analyses (SRs/MAs). Mahardawi et al systematically evaluated oral autogenous bone grafting clinical findings. Their question was: “In partly edentulous individuals, what are the alveolar ridge volume changes, histological findings, and implant durability in locations reinforced using dental autogenous bone grafting?” 15 Li et al compared the clinical and histological efficacy of autogenous demineralized dentin matrix (ADDM) as a bone graft material to Bio-Oss in strengthening oral bone lesions. 16

Bazal-Bonelli et al investigated the clinical effects of autogenous tooth root blocks on ridge reinforcement, implant survival, block absorption, postsurgical complications, and histologic outcomes. 17 Gharpure and Bhatavadekar conducted a comprehensive analysis to compare tooth bone grafts to other bone replacements in the oral and maxillofacial regions. 18 Shavit et al performed a systematic review of sinus augmentation procedures utilizing various tooth-derived bone graft materials and compared the outcomes of dental graft, xenograft, allograft, and alloplastic using radiography and histomorphometry. 19

Starch-Jensen et al conducted a systematic review of the clinical data on implant management after lateral alveolar ridge augmentation (LARA) with autogenous dental block grafting versus autogenous bone block grafting before implant placement. 20 Inchingolo et al studied engineered structures, such as tooth block grafts, growth factors, and light modulation applications for bone repair therapy. 21 Ramanauskaite et al analyzed clinical data regarding the effect of autogenous teeth on alveolar ridge augmentation. 22 Hazballa et al examined the clinical evidence of a tooth as a material for bone augmentation in alveolar ridges. 23

Based on the disparity of results in the literature, this umbrella review aims to evaluate systematic studies with clinical application results of dental bone grafting as a bone substitute in oral and maxillofacial regions.

## Method

### Study Design

The utilized articles included systematic/meta-analysis reviews and resources examining the outcomes of tooth bone grafts. The methodology adhered to the Cochrane Handbook guidelines, the Preferred Reporting Items for Systematic Reviews and Meta-Analyses (PRISMA) checklist, 18 and several high-quality methodological overviews. 19 20

### Inclusion Criteria

#### Study Type

This overview was limited to SRs/MAs of clinical trials, case controls, and cohorts on using tooth substitutes as bone grafting material in craniofacial regions.

#### Subject Type

Patients who underwent alveolar ridge grafting with a bone substitute were included, regardless of age, race, or gender.

#### Intervention Types

In the experimental group, the intervention consisted of using a tooth substitute as a grafting material for alveolar ridge/sinus augmentation. In the control group, the intervention comprised autogenous bone blocks, xenogeneic material, allogenic material, or no grafts.

#### Outcome Measurement Types

The outcome measurements included implant stability; volumetric bone changes; and clinical, histologic, radiologic, immunological, and biochemical evaluation ( Table 1 ).

**Table 1:** Inclusion and exclusion criteria and outcome evaluation of systematic reviews evaluating tooth graft

Study	Inclusion criteria	Exclusion criteria	Outcome's evaluation
Mahardawi et al 15	(1) Articles in English, (2) human studies, (3) studies reporting outcomes from six or more autogenous tooth bone graft (ATBG) grafted sites (extraction sockets or ridge defects), (4) studies where ATBG was prepared chairside and (5) was not mixed with another bone graft, and (6) studies with outcomes measured/reported at least 3 mo postoperatively	(1) Studies reporting the success of grafting procedures without quantifiable measurements, (2) publications analyzing aspects irrelevant to the focused question (e.g., probing depths or clinical attachment levels), and (3) studies with an unclear methodology, design, and/or objectives	Alveolar ridge volumetric changes after ridge preservation/augmentation, percentage of new bone formation (from reports on histological findings), marginal bone loss (MBL), implant stability quotient (ISQ), and reported complications or failures related to implants
Inchingolo et al 21	*In vivo* articles on human and animal studies in the field of craniomaxillofacial bone regeneration that highlighted the characteristics of engineered bone constructs and combinations of growth factors and photobiomodulation applications	The exclusion criteria considered for the descriptive analysis were letters to the editor and articles written in non-English languages	Not mentioned
Starch-Jensen et al 20	Studies assessing implant treatment outcome following lateral alveolar ridge augmentation (LARA) with an autogenous tooth (AT) block graft compared with autogenous bone block graft were included by addressing the previously described outcome measures. The review exclusively focused on studies using LARA with an AT block graft and lag-screw fixation prior to implant placement. In addition, at least five patients should be included, and the number of inserted implants and surgical procedures had to be clearly specified	The following exclusion criteria were applied: unspecified length of observation period, insufficient description of the surgical procedure or number of inserted implants as well as studies involving medically compromised patients. Studies assessing the autogenous dentin shell graft technique or particulate AT material in conjunction with delayed or simultaneous placement of implants were excluded as well as letters, editorials, PhD theses, letters to the editor, case reports, abstracts, technical reports, conference proceedings, cadaveric studies, animal or *in vitro* studies, and literature review papers	* * • *Survival of superstructures.* This is estimated by subtracting of failed superstructures, which is defined as a complete loss of the suprastructure due to technical and/or biological complications * * • *Survival of implants.* This is estimated by subtracting of failed implants, which is defined as mobility of previously clinically osseointegrated implants or removal of nonmobile implants due to progressive peri-implant MBL or infection * * • *Implant stability.* This is estimated by magnetic resonance frequency analysis, percussion test, or reverse torque test * * • *Health status of the peri-implant tissue (HSPIT).* Bleeding on probing, probing depth, mucosal recession, clinical attachment level, and peri-implant marginal bone level as evaluated by clinical and radiographic measurements * * • *Gain in alveolar ridge width.* This is estimated by clinical or radiographic measurements * * • *Postoperative dimensional changes of the alveolar ridge width.* This is estimated by clinical or radiographic measurements * * • *Patient-reported outcome measures* * * • *Biologic and technical complications*
Li et al 16	(1) RCT used for both randomized clinical trial and randomized controlled clinical trial (RCTs); (2) human population; (3) systemic healthy patients who suffered from oral bone defects and needed bone augmentation; (4) bone defect sites of patients in the intervention group were grafted using autogenous demineralized dentin matrix (ADDM) or ATBGs; (5) the control group was given Bio-Oss grafts; (6) at least one osteogenic function-related parameter was measured at both the baseline and follow-up time points	(1) Animal trials; (2) reviews, meta-analyses, case reports, retrospective and cohort studies, studies without a comparison group, and conference abstracts and theses; (3) no outcomes of interest; (4) duplicate studies; and (5) articles with unavailable data	The main outcomes including implant stability quotient (ISQ), sinus height (SH), the percentage of new bone formation (NBF) and residual graft material (RGM)
Bazal-Bonell et al 17	(1) Clinical human studies of alveolar ridge augmentation with autogenous tooth root block (ATRB) grafting; (2) RCTs, cohort studies, case–control studies, cross-sectional studies, and case series; (3) clinical human studies providing the following data: ATRB survival rate, bone gain, bone resorption, implant survival rates, complications, and histological findings; (4) follow-up of at least 6 mo; (5) number of patients/study arm or cohort greater than five patients; (5) articles published in English, Spanish, or German; (6) no restrictions were imposed on publication dates	Exclusion criteria were the following: (1) clinical studies carrying out any other type of ridge augmentation procedure than ATRB grafting; (2) animal studies and case reports; and (3) *in vitro* studies	The primary outcome analyzed to assess maxillary and mandibular atrophy management was the survival of the ATRB and the survival of implants placed at the augmented sites. Secondary outcomes were intra- and postoperative complications of ATRB and implants, bone gain and resorption in ATRB, changes in marginal bone levels, and histological findings. ATRB survival was considered to be the maintenance of the block in the rehabilitation area, allowing reentry surgery for implant placement. For dental implants, survival was understood as no mobility, no progressive MBL, or infection leading to implant removal
Hazballa et al 23	Study in craniomaxillofacial bone, human study, and publication of the last five	Study in animal, *in vivo* and *ex vivo*	Histological outcomes, NBF, and ridge dimension loss
Shavit et al 19	* * • Studies including at least 5 subjects (animal or human) undergoing sinus augmentation. * * • Studies evaluating the use of tooth-derived graft materials in the sinus augmentation procedure * * • Studies evaluating the tooth-derived graft material by at least one of the following diagnostic tools: panoramic radiography, computed tomography, histological or histomorphometric analysis * * • Studies that assessed the tooth-derived graft material by comparing to control groups receiving sinus grafting with other materials such as xenograft, allograft, and alloplast * * • Follow-up period of at least 4 mo after grafting for human subjects and 8 wk for animals	* * • Studies in which autogenous tooth was mixed with autogenous bone to graft the maxillary sinus * * • Studies that evaluated human patients without mentioning their medical condition * * • Technical notes * * • Case reports	Gained bone height after grafting, stability of the sinus graft height, bone formation, and regeneration potential as shown in the histomorphometric analysis, implant stability, complications, and implant survival. NBF (%) RGM (%) Marrow space (%) Osteoid thickness (µm)
Ramanauskaite et al 22	* * • RCTs, controlled clinical trials (CCTs), prospective/retrospective observational studies, or prospective/retrospective case series with a minimum of 10 patients (5 per group in controlled studies) in good general health who needed implant therapy and bone reconstruction procedures * * • Studies in which lateral ridge augmentation procedures and/or alveolar ridge contour augmentation (i.e., procedures aimed at increasing the ridge volume beyond the skeletal envelope existing at the time of extraction) and/or maxillary sinus floor elevation and/or augmentation of extraction sockets were performed using AT and/or materials other than AT (in controlled studies) at the time of implant placement (simultaneous) or prior to implant placement (staged) * * • ATs used as a block or in particulate form with or without additional grafts or barrier membrane * * • Studies reporting on the specified primary or secondary treatment outcome	* * • Case reports and studies with unclear designs * * • Animal studies * * • Studies assessing the efficacy of interventions aimed at preserving extraction sockets (i.e., procedures aimed at preserving the ridge volume within the envelope existing at the time of extraction) * * • Studies including patients with compromised systemic health	Alveolar ridge augmentation *Primary outcomes:* Alveolar ridge width Cone beam computed tomographic (CBCT) analyses Bone gain at dehiscence-type peri-implant defects *Secondary outcomes:* Postoperative complications Graft resorption Feasibility of implant placement Implant stability Sinus floor elevation Extraction socket augmentation Surgical intraoperative complications Feasibility of implant placement after the healing period
Gharpure and Bhatavadekar 18	(1) RCT, clinical cohort study, or a well-designed CCT, (2) assessed treatment of tooth bone graft, (3) statistically compared tooth bone graft with ungrafted controls or other graft materials (not applicable for clinical cohort), (4) reported clinical, histologic, radiologic, immunologic, or biochemical results	Studies were excluded if they used additional materials mixed with tooth bone graft. Studies that were not published in peer-reviewed PubMed indexed journals were also excluded	* * • *Clinical:* removal torque value (RTV), ISQ, change in pocket depth, vertical and horizontal dimension of the alveolar bone * * • *Histologic:* mean new bone formation volume (MNBFV), mean new bone formation area (MNBFA), trabecular bone area (TBA), new bone formation rate (NBFR), bone implant contact (BIC), crestal width (CW), and augmented area (AA) * * • *Radiologic:* optical density (OD), bone volume/tissue volume (BV/TV) on CT and micro-CT, bone mineral content (BMC), bone density increase (BDI), mean new bone density (MNBD), vertical and horizontal dimension of the alveolar bone, mean increase in bone height and mean resorption in bone height * * • *Immunological:* vascular endothelial growth factor (VEGF), angiogenesis percentage (A-P), immunostained osteoblast number (IO-N) labeled for bone morphogenic proteins 2 and 4 (BMP-2 and BMP-4) * * • *Biochemical:* alkaline phosphatase activity (ALP-A), osteocalcin antigen activity (OC), and microassay for BMP-2, BMP-4, COL1A, RUNX, and CCL2 levels

In addition, the following criteria were applied to the articles: (1) systematic reviews of at least two primary studies; (2) a focus on the outcomes of using tooth substitutes as bone graft material in human alveolar ridge/sinus augmentation, (3) systematic reviews comprising longitudinal studies examining the impact of use of tooth substitutes for orofacial bone augmentation and reporting at least one type of histological/radiological/clinical outcomes; and (4) publications in English.

We excluded the following: (1) systematic reviews that included only animal studies, (2) systematic reviews that included the use of tooth substitutes for grafting other parts of the human body besides the orofacial region, (3) systematic reviews or meta-analyses with unclear inclusion/exclusion criteria and (4) fully overlapping datasets, and (5) studies that report results other than those of interest.

A search was conducted in the electronic databases PubMed, MEDLINE, EMBASE, Cochrane library, and Google Scholar for articles published up until August 15, 2022, considering language restrictions (only English) and PRISMA guidelines. The search was performed using the Mesh medical subject heading and nonmesh terms in simple or multiple conjunctions. The following keywords were used: (dent*) AND (autogen*) AND (“autogenous tooth bone graft” OR ATBG OR autogen OR tooth graft OR autolog OR ADDM OR allogenous OR xenogenous). These filters were applied to the search results: “systematic review” and “meta-analysis.”

### Data Extraction

**Fig. 1:**
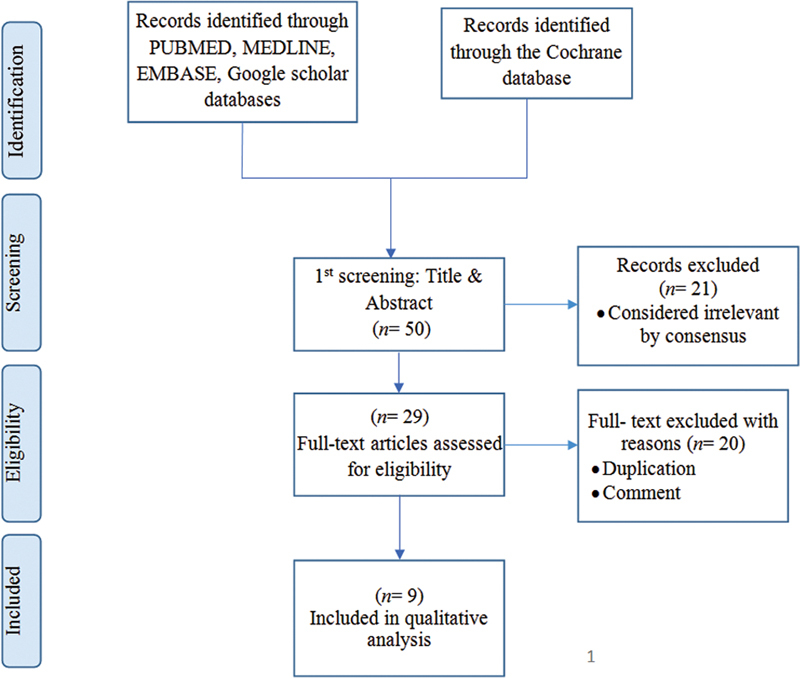
Flowchart for the studies identified, screened, and included in the study.

**Fig. 2:**
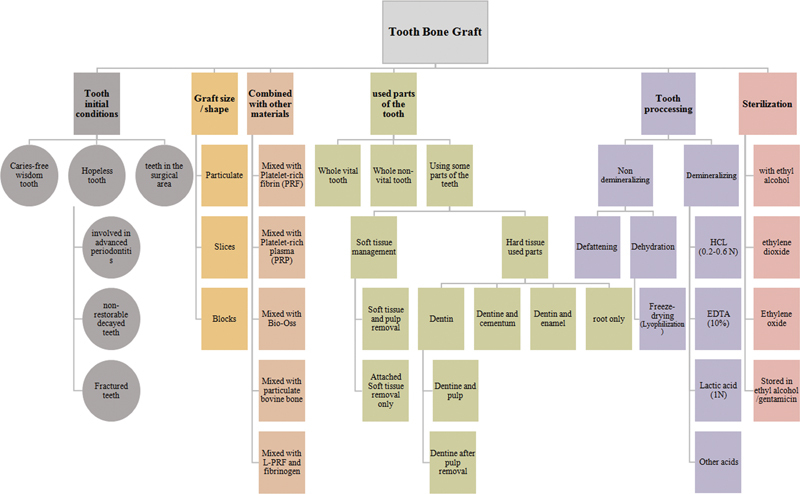
A ﬂow diagram of the different methods of tooth bone graft preparation used is presented.

Fig. 1 depicts the data extraction procedure utilized in this study. Fig. 2 summarizes the different methods and conditions used for preparation of tooth bone graft material reviewed by SRs/MA.

Table 2 details the main characteristics of the studies included. Data extraction from eligible publications was based on systematic/meta-analysis review reports for the tooth bone graft outcomes. Studies were included if they focused on tooth grafting and were in English. Abstracts, review articles, editorial articles, guidelines/protocols, and articles not published with appropriate clinical outcomes were excluded from the study.

**Table 2:** Baseline characteristics of systematic reviews evaluating tooth graft

Study	Types/no. of studies included	No. of patients	Analysis method	Search period	Population	Interventions	Comparison	Main results	Risk of bias	Review quality
Mahardawiet al 15	9 RCTs 5 P 6 R	119	SR	Up to May 2021	Patients with alveolar bone defects resulting from tooth extraction/tooth loss who require bone augmentation and implant placement	The use of chairside-prepared autogenous tooth bone graft (ATBG) for bone augmentation and implant placement	The primary outcome was the postoperative volumetric changes in alveolar ridge dimensions, and the secondary outcomes were the implant survival rate at the final follow-up and the percentage of new bone formation in the graft area as determined by harvesting core biopsies	Investigations revealed evidence that autogenous bone grafting using prepared teeth could be as effective as other bone grafting materials	Low	High
Inchingolo et al 21	304	–	SR	Up to May 2021	304 articles met the inclusion criteria. Based on the bone substitutes utilized, these articles were divided into alloplastic, autologous, xenograft, platelet-derived, laser therapy, microbiota, and mesenchymal cell groups	Explore recent advances in bone repair as they relate to the therapeutic potentials of engineered structures, growth factors, and light modulation applications	Investigate new achievements in bone repair concerning therapeutic potentials based on engineered structures, growth factors, and light modulation applications	Due to the long-term clinical benefit to the oral microbiota and the patient's systemic health, it is possible to use biocompatible and resorbable bone substitutes for bone reconstruction. In addition, growth factors can reduce concurrent diseases of the repair process and enhance the recovery phase following surgery	Low	High
Starch-Jensen et al 20	3 P	65	SR/MA	Up to December 2021	Healthy patients with a horizontal alveolar deficiency following tooth loss, trauma, or congenitally missing tooth/teeth	Lateral alveolar ridge augmentation with an autogenous tooth block graft	Lateral alveolar ridge augmentation with an autogenous bone block graft	A possible alternative to a bone block is the use of a dental block to reinforce the lateral alveolar ridge	Low	High
Li et al 16	7 RCTs	220	SR/MA	Up to July 2021	Patients with oral bone defects	Autogenous demineralized dentin matrix (ADDM) as bone graft material	Bio-Oss	ADDM could be as effective as Bio-Oss in bone augmentation for oral bone defects	Low	High
Bazal-Bonelli et al 17	5 P 2 R	136	SR	Up to December 2020	Systemically healthy edentulous and partially edentulous patients	A comparison of autogenous tooth root blocks (ATRBs) for alveolar ridge augmentation at dental implant sites	Survival rate and complications of ATRB and implants placed in augmented sites; bone gain and bone resorption of ATRB, and histological results at graft sites	The reconstruction of alveolar crests using ATRBs may be a viable option for single-tooth loss and low levels of bone atrophy in terms of block survival and subsequent implant placement	Low	High
Hazballa et al 23	3 P 2 Pilot	95	SR	2017–2021	Human patients with craniomaxillofacial sites defects	The use of tooth graft in alveolar ridge preservation	Anorganic material, bovine bone, the endodontic treated tooth	An autologous tooth matrix is a bioactive scaffold that paves the way for new developments in bone regeneration. Autologous tooth matrix is a promising material for the preservation of ridges	Low	High
Shavit et al 19	2 P 2 R 3 AC	136	SR	Up to March 2019	Human patients with atrophied posterior maxilla undergoing sinus elevation with bone graft materials produced from teeth	Sinus augmentation using tooth material in different forms: powder, block	Other bone graft materials used in sinus augmentation: allograft, xenograft, and alloplast	Tooth-derived graft materials such as xenograft, allograft, and alloplast could be successful in sinus augmentation procedures	Low	High
Ramanauskaite et al 22	3 RCTs 2 P 1R	149	SR	Up to March 2018	Patients exhibiting alveolar ridge deficiencies and needing an implant-retained restoration	Reconstructive procedures employing autogenous teeth (AT) vs. reconstructive procedures employing materials other than AT	Reconstructive procedures employing materials other than AT	AT can be proposed as an alternative material for reconstructing alveolar ridge deficiencies and be effective in the clinic	Low	High
Gharpure and Bhatavadekar 18	4 RCTs 1 R 3 P 18 AC	184	SR	Up to January 2017	Humans with a tooth bone graft in the oral and maxillofacial region	The efficiency of the tooth bone graft used as a bone substitute	Ungrafted sites and/or sites grafted with other bone substitutes, as determined by clinical, histologic, radiologic, immunologic, and biochemical analysis	Tooth bone grafting offers no advantages beyond those of conventional grafting materials. However, nonstandard processing and heterogeneity of study results restrict its clinical application	Low	High

Abbreviations: AC, animal clinical; P, prospective; R, retrospective; RCT, randomized clinical trial; SR/MA, systematic review/meta-analysis.

Two independent reviewers (M.R. and Q.P.) determined which studies were eligible for analysis (1.0 kappa). One researcher (M.R.) was responsible for extracting qualitative or quantitative data from the studies, while the second researcher (Q.P.) was responsible for validating all qualified information. Information collected included the author's name, the year and type of study, the number of patients, an evaluation of outcomes, a comparison of results, and a conclusion.

### Data Analysis

There were significant differences between eligible articles regarding participant inclusion criteria, populations, alternative procedures, and follow-up duration ( Table 2 ), indicating a high degree of heterogeneity among studies. As a result, it is unreasonable to assume that all reviews assessed the same effect. 24

### Bias Risk Assessment

**Fig. 3:**
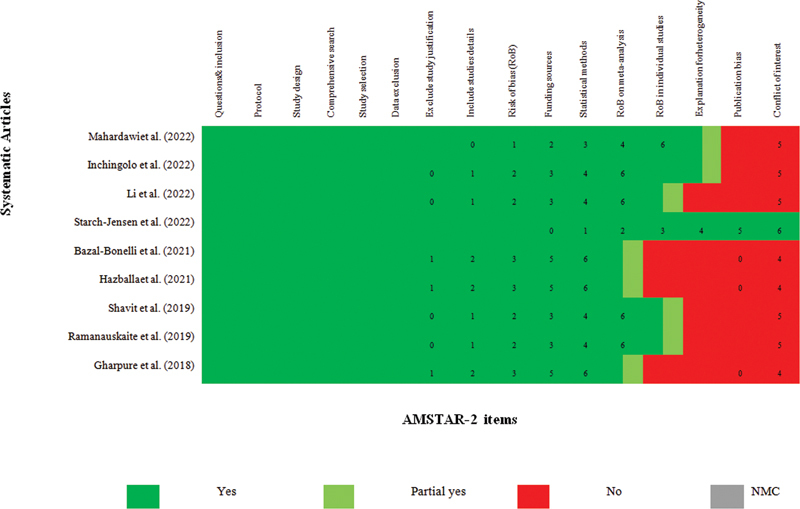
The Assessment of Multiple Systematic Reviews 2 (AMSTAR2).

We used 16 questions from the Assessment of Multiple Systematic Reviews 2 (AMSR2) 25 tool to assess the quality of systematic/meta-analysis review studies based on the risk of bias assessment ( Fig. 3 ). Each article was ultimately assigned a score that indicates the likelihood of bias in the study. If 8 to 11 questions were answered positively, the risk of bias was low; if 4 to 7 questions were answered positively, the risk of bias was moderate. The risk of bias was deemed high if fewer than three questions received positive responses. 26 Two qualified researchers assessed the articles (kappa = 0.90). Inconsistency and ambiguity were resolved through discussions. The third investigator assisted in resolving unresolved issues.

## Results

### Screening of Systematic/Meta-Analysis Reviews

The PubMed, MEDLINE, EMBASE, Cochrane library, and Google Scholar databases yielded 50 systematic/meta-analysis review articles. After removing duplicate sources, the titles and abstracts of 29 studies remained to be examined. After thoroughly evaluating these publications, 21 met the eligibility criteria, and their full papers were read. Finally, nine review articles 15 16 17 18 19 20 21 22 23 were selected for data extraction in the present umbrella study. Fig. 1 and Table 2 detail the research strategy and summarize the most important characteristics of the articles. Regarding the inclusion and exclusion criteria and evaluation of outcomes, the included SRs exhibited a wide range of approaches ( Table 1 ). There was only a slight overlap between the reviews that utilized tooth grafts ( Table 3 ).

**Table 3:** Primary included studies included in the systematic reviews for using the tooth material as bone grafting material

Study	Gharpure and Bhatavadekar 18	Shavit et al 19	Starch-Jensen et al 20	Bazal-Bonelli et al 17	Mahardawi et al 15	Li et al 16	Inchingolo et al 21	Ramanauskaite et al 22	Hazballa et al 23
Andrade et al 27					**×**				
Chung and Lee 28	×								
Del Canto-Díaz et al 29					×		×		×
Elfana et al 30					×				
Gomes et al 31	×								
Jeong et al 32		×							
Lee et al 33								×	
Jin et al 34						×			
Joshi et al 10	×				×				
Jun et al 35	×	×				×		×	
Jung et al 36						×			
Kim et al 37				×					
Kim et al 38	×	×						×	
Kim et al 39				×	×				
Kim et al 40		×				×			
Kim et al 41				×					
Lee and Kim 42	×	×							
Li et al 43					×	×			
Melek and El Said 44					×				
Minamizato et al 45					×				
Minetti et al 46					×		×		×
Minetti et al 47							×		
Minetti et al 48					×				
Minetti et al 49							×		
Minetti et al 50					×				
Movin and Borring-Møller 14	×								
Pang et al 9	×					×		×	×
Parvini et al 51				×					
Parvini et al 52			×		×				
Pohl et al 53					×				
Radoczy-Drajko et al 54					×				
Sánchez-Labrador et al 55					×				
Santos et al 56						×			
Schwarz et al 57				×	×			×	
Schwarz et al 58				×	×			×	
Schwarz et al 59			×		×				
Shejali et al 60				×					
Sohn and Moon 61		×							
Valdec et al 62							×		×
Wang et al 63			×						
Wu et al 64					×				
Xiao et al 65				×					
Xu et al 66		×							
Yüceer-Çetiner et al 67					×				

### Evidence Quality

AMSR2 was used to evaluate the risk of bias. The level of bias in the study was classified as high, medium, or low based on the number of correct answers ( Fig. 3 ). The risk of bias in this study (including all systematic and meta-analysis reviews) was low. Clinical evidence consisted of articles with a low risk of bias. To this end, low-risk studies accounted for 100% of the study volume ( Fig. 3 ).

### Characteristics of Systematic Reviews

Tables 1 and 2 provide general information about each systematic/meta-analysis review. In addition, authors and publication year, number and type of studies, type of analysis, interventions, outcomes, bias risk, key findings, inclusion and exclusion criteria, and outcome evaluation of each SR/MA are reported. Fig. 2 summarizes different methods and conditions used for preparation of tooth bone graft material reviewed by SRs/MA.

Mahardawi et al conducted a systematic review that included 20 studies. 15 Li et al systematically searched eight databases for RCT studies. The included studies' quality was assessed using the Cochrane Collaboration's risk tool, and the data were analyzed using Stata 15.0 software. 16 PRISMA guidelines were used in the study of Bazal-Bonelli et al, and the search was conducted in four databases. The Newcastle-Ottawa Quality Assessment Scale 17 was used to assess the quality of the selected studies.

In the systematic review conducted by Gharpure and Bhatavadekar, a search was conducted to identify animal and human clinical studies and their risk of bias. 18 In the systematic study by Shavit et al, a database search was conducted to identify articles on dental bone grafting in sinus augmentation. 19 In a systematic review by Starch-Jensen et al, searches were conducted in three databases. The Cochrane risk of bias tools, the Newcastle-Ottawa scale, and the GRADE system were utilized to evaluate the quality of included studies. 20 In the research conducted by Inchingolo et al, four databases were queried. 21 In the Ramanauskaite et al systematic review, six studies met the criteria for inclusion. Autogenous teeth were utilized in lateral reinforcement, demineralized dentin matrix, vertical reinforcement of sockets after extraction, and height of the lateral sinus floor. 22

In the systematic review by Mahardawi et al, alveolar bone dimensions were reported for heights ranging from –0.64 to +2.26 mm and widths ranging from –1.21 to +0.41 mm. In addition, a significant increase in the dimensions of the additional sites was observed. The survival rate was 98.8% for delayed implant placement and 97.4% for immediate implant placement. Furthermore, utilizing this graft increased the percentage of ossification and bone volume at different time points after surgery. 15

Li et al performed a systematic review of seven RCTs involving 220 patients. These studies observed an insignificant difference in the amount of new bone formation or implant stability. In addition, sinus height and the percentage of residual graft material were significantly lower in patients who received ADDM grafts than in those who received Bio-Oss grafts. 16

In the review by Bazal-Bonelli et al, seven studies involving 136 patients met the inclusion criteria and were examined. Among the patients, 118 were treated with autogenous tooth root blocks (15.99% survival), and 26 were treated with autogenous bone blocks (100% survival). The average bone formation in tooth root blocks was comparable to autologous bone formation. Additionally, the implant survival rate in autogenous tooth root blocks was 98.32%. 17

In the systematic review by Gharpure and Bhatavadekar, the inclusion criteria were met by 18 animal-controlled and 8 human RCTs involving 184 patients. High levels of heterogeneity existed between the selected studies. In more than half of the studies (71.42% of clinical studies and 55.56% of animal studies), tooth and bone grafting did not demonstrate a statistically significant difference between the tooth bone graft and the control group. In addition, 50% of clinical trials and 63.33% of animal studies posed a low risk of bias. Moreover, 350 patients exhibited adverse effects (18.86%). 18

Seven articles met the inclusion criteria for the systematic study by Shavit et al. Data extraction was based on the diagnostic tool type, which included residual alveolar height, increased graft height, and absorption height. 19

In a review conducted by Starch-Jensen et al, three studies met the criteria for inclusion. Comparing these studies revealed no statistically significant differences between the two treatment methods regarding short-term implant survival, the health of the tissue surrounding the implant, or the occurrence of complications. In contrast, the tooth block resulted in a significantly narrower alveolar ridge than the bone block. However, short-term mucositis was reported more prevalent around dental implants with dental blocks. 20

In the survey performed by Inchingolo et al, 304 articles met the criteria for inclusion. Based on the bone substitute utilized, these articles were divided into alloplastic, autologous, xenograft, platelet-derived, laser therapy, microbiota, and mesenchymal cell groups. 21

Hazballa et al concluded that a dental matrix is a viable option for autologous transplantation because it does not cause an antigenic reaction, permits three-dimensional bone reconstruction, is simple to prepare, and is cost-effective. 23

## Discussion

This umbrella review investigates available evidence regarding using tooth substitutes as bone grafting material in the oral and maxillofacial regions. Due to the availability of numerous recent SRs, it became evident that an umbrella review approach should be adopted to avoid duplication of available evidence and to cover all tooth graft techniques comprehensively. This approach was also supported by the fact that most published SRs considered a variety of tooth graft preparation or outcome evaluation techniques. This heterogeneity prevents most of them from conducting a meta-analysis to determine the extent of this graft's effectiveness. In this umbrella review, 9 15 16 17 18 19 20 21 22 23 systematic/meta-analysis articles comprising 81 (23 RCTs and 58 non-RCTs) articles were included. The outcomes extracted from these umbrella studies were associated with tooth graft procedures.

All included studies stated that additional long-term research is necessary to confirm their findings. Only one systematic review, which included a meta-analysis, compared the clinical outcomes of demineralized dentin grafts and Bio-Oss and concluded that they are equally effective for augmenting oral bone defects. 16 A systematic review on tooth root blocks concluded that reconstruction of alveolar crests using autogenous tooth root blocks appears to be an adequate solution for single-tooth gaps and low grades of bone atrophy in terms of the survival of the bone block and the implants placed afterward. 17

According to several review articles, the tooth bone graft offers no additional benefits over other graft materials. In addition, the lack of predictability regarding resorption time and clinical success and the lack of standard processing methods restrict the use of this material in clinical practice. 18 Others have demonstrated that the bone formation capacity of autogenous tooth graft is comparable to or greater than that of Bio-Oss or the combination of allograft and xenograft, particularly when used in the maxillary sinus. 19 Following LARA with autogenous tooth block graft, studies indicate a high short-term implant survival and implant stability index. After 26 weeks of functional implant loading, the peri-implant tissue health following LARA with the two treatment modalities was reported to be comparable. However, a higher incidence of short-term peri-implantitis was observed after LARA with autogenous tooth block grafting. 20

The most definitive data for evaluating the osteogenic capability of bone graft material 16 were derived from the histological analysis of human tissue specimens harvested from graft sites. Nevertheless, each study provides a variety of outcome evaluations, such as clinical, radiological, and histological outcomes. Regarding the follow-up duration, SRs did not apply any limitations and the included primary studies showed a variety from 2 weeks to 4 years of follow-up (mostly 6 months). Due to its close proximity to the implant surface, 68 69 the dental matrix shows promise as a biomaterial for preserving the alveolar ridge. The initial condition of the extracted tooth, its combination with other materials (Bio-Oss, Platelet-rich plasma (PRP), Platelet-Rich-Fibrin (PRF)), the size and shape of the particles (ground, sliced, or in block form), treatment (materials used for demineralization and sterilization), composition (enamel, dentin, cementum, pulp, and soft tissue), and graft dimensions were not standardized among the selected studies.

In addition, except for one study 19 that focused solely on sinus augmentation, other studies did not specify the location of the augmented alveolar bone. Inadequate homogeneity and standardization in the processing of graft material make comparisons with other materials more difficult and diminish the graft material's clinical value. In conclusion, it is evident that the reviewed articles displayed significant heterogeneity and that the original studies are inconclusive and require additional research on this topic. However, given the risks of transplant rejection and the difficulty of obtaining autogenous graft material, tooth grafting can be recommended with caution.

## Conclusion and Recommendation

Autogenous tooth bone grafts appear to be effective in oral defect reconstructions compared to Bio-Oss, autogenous bone blocks, or no-grafts, despite the heterogeneity of the included SRs and primary studies, as confirmed by the results of the current umbrella overview. There is some evidence that these techniques improve clinical, histological, or radiological outcomes; however, additional research is needed to inform guideline development and to ensure that treatment recommendations are based on long-term clinical outcomes. There is a clear need to increase the emphasis on using tooth bone grafts in managing alveolar ridge resorption as a mainstream option, as the conventional approaches such as Bio-Oss or autogenous bone blocks are prohibited due to cost or surgical site morbidity.
